# Effect of the 13-valent pneumococcal conjugate vaccine on nasopharyngeal carriage by respiratory pathogens among Greenlandic children

**DOI:** 10.1080/22423982.2017.1309504

**Published:** 2017-05-03

**Authors:** Johan Emdal Navne, Anders Koch, Hans-Christian Slotved, Mikael Andersson, Mads Melbye, Karin Ladefoged, Malene Børresen

**Affiliations:** ^a^ Department of Epidemiology Research, Statens Serum Institut, Copenhagen, Denmark; ^b^ Department of Microbiology and Infection Control, Statens Serum Institut, Copenhagen, Denmark; ^c^ Department of Internal Medicine, Queen Ingrids Hospital, Nuuk, Greenland; ^d^ Department of Pediatrics, Rigshospitalet, Copenhagen, Denmark

**Keywords:** PCV-13, carriage, Inuit, respiratory infections, pneumococcus

## Abstract

**Background**: In 2010, Greenland introduced the 13-valent pneumococcal conjugate vaccine (Prevnar 13®– PCV-13) in the childhood immunisation program. The authors aimed to evaluate the impact of PCV-13 on nasopharyngeal carriage of bacteria frequently associated with respiratory infections in children.

**Method**: In 2013 a cross-sectional population-based study of nasopharyngeal carriage was conducted among Greenlandic children aged 0–6 years and results were compared with an equivalent study from 2011. Nasopharyngeal swab samples were tested for *Streptococcus pneumoniae*, non-typeable *Haemophilus influenzae*, *Moraxella catarrhalis* and *Staphylococcus aureus*. Pneumococcal serotyping was performed by Quellung reaction and serotype-specific antisera. Statistical analysis included logistic regression models, adjusting for known risk factors.

**Result**: A total of 377 nasopharyngeal samples were collected. Overall carriage rate of *S. pneumoniae* remained unchanged from 2011 to 2013 (51% and 56%, p=0.13), but significant serotype shifts were observed among both vaccinated and unvaccinated children with marked reductions in carriage of vaccine-type pneumococci, counterbalanced by increasing carriage of non-vaccine types. Carriage rate of *S. aureus* decreased significantly among vaccinated children whereas that of *M. catarrhalis* increased.

**Conclusion**: PCV-13 introduction in Greenland is associated with significant changes in nasopharyngeal bacterial carriage. Continued surveillance is warranted to clarify whether these changes are persistent, and affect the pattern of respiratory and invasive diseases in Greenland.

## Introduction

The Inuit population of Greenland suffers from high rates of respiratory infections and invasive bacterial diseases [1], causing higher morbidity and mortality than among non-Inuit [1–7].

Nasopharyngeal bacterial carriage is the essential precursor of these infections with *Streptococcus pneumoniae*, non-typeable *Haemophilus influenzae* (NTHi), *Moraxella catarrhalis*, and S*taphylococcus aureus* as the most clinically relevant bacteria [8–11]. Besides being a source of respiratory infections, nasopharyngeal colonisation is also a source of transmission to other individuals. Greenlandic children are frequently colonised by these agents, with acquisition shortly after birth and frequent co-colonisation by multiple species [12]. The disease burden from respiratory tract infections in Greenlandic children is among the highest reported [4], primarily in the form of acute and chronic otitis media with frequent long-term sequelae [13]. The Inuit populations of Alaska and Canada share the high risk of respiratory infections as well as invasive pneumococcal disease (IPD) [2,14,15]. In Greenland, Inuit have four times as high risk of invasive pneumococcal disease compared with non-Inuit [16].

The 7-valent pneumococcal conjugate vaccine (Prevnar 7®; Pfizer/Wyeth (PCV-7)), introduced in the childhood immunisation program in 2000 in the US and gradually widespread in many countries, has proven efficient in reducing disease rates caused by the 7 serotypes included in the vaccine (4, 6B, 9V, 14, 18C, 19F and 23F), so-called vaccine types (PCV-7 VT) [17]. Furthermore, the PCV-7 has proven to be able to prevent PCV-7 VT nasopharyngeal carriage and thus interrupt the chain of transmission to other individuals including non-vaccinated persons, resulting in herd immunity [18]. However, the overall effect on pneumococcal carriage rates has been somewhat counterbalanced by increasing rates of pneumococcal serotypes not included in the vaccine (non-vaccine types: PCV-7 NVT), a phenomenon known as replacement [17,19,20]. Recent studies indicate that the carriage rates of other co-colonising pathogens such as *S. aureus* and NTHi may also be changed after PCV introduction [18,21]. These observations have subsequently raised the concern of an altered disease pattern after widespread use of PCV, given the polymicrobial pathogenesis of respiratory diseases. Thus, in a randomised controlled trial from Finland, higher rates of acute otitis media (AOM) caused by NTHi were observed among PCV-7 vaccinated children compared with a control group of unvaccinated children [22]. Also, *S. aureus*-related AOM has been observed in higher proportions among those who are PCV-7 vaccinated [23]. A possible mechanism for the observed changes may be the dynamic nature of the nasopharyngeal microbial composition, where the balance may be skewed due to a PCV-related clearance of vaccine-type (VT) pneumococci which leaves the nasopharyngeal niche vacant to be occupied by other opportunistic microbes [24].

In 2000 the PCV-7 was introduced in the childhood immunisation program in Alaska. In the following years the overall incidence rate of IPD in the total childhood population of Alaska was reduced, but among native Alaskans, a dramatic increase in IPD rates caused by non-vaccine types (NVT) resulted in an even bigger disparity in IPD rates between the Native Alaskan and the general US population [25]. In 2010 in Alaska the PCV-7 was replaced by the 13-valent pneumococcal conjugate vaccine (PCV-13). This resulted in further reductions in IPD rates, particularly those caused by PCV-13 serotypes (1, 3, 4, 5, 6A, 6B, 7F, 9V, 14, 18C, 19A, 19F and 23F), but also in rates of IPD caused by non-PCV-13 serotypes [26]. However, the decrease in non-PCV-13 IPD rates may not be sustained, as a carriage study from 2014 in Alaskan natives found increasing rates of non-PCV-13 carriage in the years after PCV-13 introduction [27].

Greenland introduced PCV-13 (Prevnar 13®; Pfizer/Wyeth) in the childhood immunisation program in September 2010 [28]. We aimed to describe possible changes in nasopharyngeal carriage rates by four clinically important bacteria among Greenlandic children aged 0–6 years from 2011 to 2013 following the introduction of PCV-13 in Greenland.

## Methods

We conducted a cross-sectional carriage study from October to December 2013 and compared results with those from a previous cross-sectional study conducted between October and December 2011 [12]. Since 1972 all citizens of Greenland have been given a unique personal identification number registered in the Civil Registration System (CRS). The daily updated CRS contains vital information about date and place of birth, gender, birth order, siblings, parents, and current and earlier addresses [29]. The unique personal identification number allows for accurate linkage between all national registers. Through the CRS registry, we identified all children aged 0–6 years living, in October 2013, in the towns of Tasiilaq (East Greenland) or Sisimiut (West Greenland) including their surrounding settlements, and invited the children via their parents to participate. After giving informed consents, parents or caretakers completed a questionnaire with help from an interpreter regarding birthplace, number of siblings, day-care institution attendance, recent antibiotic use, domestic tobacco exposure, recent respiratory tract infections (otitis media, tonsillitis, pneumonia or ear-discharge within the last 3 months or rhinitis within the last week), hospitalisations within the last 3 months, number of rooms in household exclusive of bathroom and kitchen, and number of children less than 5 years sleeping in the same room. Ethnicity was based on the parents’ place of birth. If both parents were born in Greenland, ethnicity was defined as Inuit; one parent born in Greenland and one elsewhere or unknown was defined as a “mixed Inuit”, and no known parents born in Greenland as “other”, which is mainly Danish (Caucasian) ethnicity. Data on PCV-13 doses were obtained from local medical files. The PCV-13 is administered as a 2+1 schedule given at 3 and 5 months of age, with a booster at 12 months of age. During the introduction of the vaccine, a catch-up campaign was initiated, offering three vaccinations for children aged 3–11 months, and two vaccinations for children aged 12–23 months [28].

### Ethical approval

The study fulfilled the Helsinki II Declaration and was scientific ethically approved by the Greenlandic Scientific Commission (Journal no. 2011 – 056257, doc. no. 738293, Journal no. 2012 – 060783, doc.no. 821618) and the Danish Data Protection Agency (2008 – 54-0427).

### Nasopharyngeal sampling

Children were sampled at the local day-care institutions and schools or invited to the health-care centre to participate. Briefly, the World Health Organisation protocol for pneumococcal carriage studies [30] was followed, sampling from the posterior nasopharynx with Minitip Flocked Nylon Swabs (FLOQSwabs™, Copan, Italy) and using Skim milk-Tryptone-Glucose-Glycerin medium (STGG), which has proven useful for the study of respiratory pathogens including *S. pneumoniae*, NTHi and *M. catarrhalis* [31]. Samples were temporarily stored (up to 3 weeks) at –20°C before shipping to Denmark for storage at −80°C. We used the same procedure as was used in the study from 2011 [12].

### Laboratory analysis

Swab samples were thawed and tested at Statens Serum Institut (SSI), Copenhagen, Denmark, for the presence of *S. pneumoniae*, NTHi, *M. catarrhalis* and *S. aureus* using standard culture methods. Bacterial identification was based on colony morphology by conventional microbiologic procedures and verified by MALDI/TOF mass spectrometry [32].

Pneumococci were identified based on α-hemolysis, optochin sensitivity and capsular reaction (Quellung). Non-typeable pneumococci were identified using a bile solubility test. Pneumococcal group-determination was performed directly on the serum-broth-enriched nasopharyngeal samples by Pneumotest latex® (SSI, Denmark) agglutination and serotypes identified with Quellung [33] reaction by the use of type-specific antisera from SSI [34,35]. To increase the likelihood of detecting low-density carriage and multiple pneumococcal serotype carriage, we added 50 µl of the nasopharyngeal swab samples to a 2 ml serum-ox broth and incubated in CO_2_, 37°C for 24 hours, before plating. This has previously been shown to increase the detection level of *S. pneumoniae* in nasopharyngeal swab samples [35].

*M. catarrhalis* was isolated from chocolate-agar plates containing antibiotics (Polymyxin B (27246 IE), Lincomycin (0.001 gr), Amphotericin B (0.002 gr) and Trimethoprim (0.003 gr)) to inhibit the growth of most Gram-positive and Gram-negative bacteria as well as fungal species. We identified the bacteria by their morphology and typical characteristics of *M. catarrhalis*, such as being able to slide the colonies along the agar surface without disrupting them (“the Hockey-puck sign”). *S. aureus* were isolated from blood-agar plates by their morphologic appearance (yellow or white colonies) and tested with a catalase test and if positive by coagulase testing using Staphaurex Plus® (OXOID). Subsequently the different species were verified by MALDI/TOF.

### Statistical analyses

The study was designed to demonstrate potential changes in pneumococcal carriage after the introduction of the PCV-13 as the primary outcome, and changes in NTHi, *M. catarrhalis* and *S. aureus* as secondary outcomes. Based on the most recent figures from Greenland in 1996 [36], carriage rates in children aged 0–7 years were estimated to be 67, 42 and 8% for NTHi, *M. catarrhalis* and *S. aureus*, respectively. Given these estimates sample sizes of 350 in each of the 2 years (2011 and 2013) would be sufficient to detect a minimal difference in carriage between the years of 10, 11 and 7%, with 80% power and a significance level of 5%. Differences in carriage rates were tested by chi-square tests, with a p-value <5% considered significant. Associations between nasopharyngeal carriage of the four bacteria and PCV-13 vaccination status were estimated by multivariable logistic regression analyses. Possible confounders were tested separately in a basic model adjusted for age (1-year intervals), sex, PCV-13 vaccination status and sampling-year. A final multivariate model was constructed by including all possible confounders significant in the basic models. This included age, sex, PCV-13 vaccination status, sampling-year, ethnicity, region (East/West), current day-care attendance, having siblings in day-care and recent respiratory infections. Statistical analyses were performed using SAS version 9.4 (SAS Institute, Inc., Cary, North Carolina). For analytical purposes we grouped pneumococcal serotypes into vaccine types (VT) and non-vaccine types (NVT). In case of potential repeated measurements from individuals appearing in both of the two cross-sectional studies, we did a robustness analysis using a general estimation equation [37] to account for correlated data.

## Results

A total of 377 children participated in 2013 with demographic characteristics as shown in Table 1. Eighty-six children also participated in the previous cross-sectional study from 2011 [12]. When testing the effect of repeated measurements on bacterial colonisation only minimal changes in estimates and confidence intervals were observed, and the interpretation of results was unchanged. The distribution of most baseline characteristics of the study population in this study was comparable to those of the previous study from 2011 (Table 1). However, significant differences were seen with regard to proportions of the children sampled in East and West Greenland, day-care attendance, in-house smoking and fractions of PCV-13 vaccinated children.

**Table 1.** Demographic characteristics of study population 2013 (n=377 children) and the population from a comparable cross-sectional study in 2011 (n=352) [12].

Variable	Level	Year 2013 n=377 (%)	Year 2011 n=352 (%)	p^a^
Gender	Male	192 (51)	181 (51)	0.98
Age, years	Median (Q1;Q3)	3.50 (1.68; 5.12)	2.85 (1.08; 4.84)	0.34
Ethnicity	Inuit	343 (91)	324 (91)	0.35
Region of Greenland^b^	East-coast	186 (49)	122 (35)	**<0.001**
	West-coast	191 (51)	230 (65)	
Day-care attendance (current)	+	232 (62)	258 (73)	**<0.001**
Having siblings in DC^c^	+	134 (36)	137 (39)	0.29
In-house smoking^d^	+	39 (10)	83 (24)	**0.002**
Sharing bedroom with children <5 years of age	+	210 (56)	210 (60)	0.45
Number of persons per room^e^	0	23 (6)	14 (4)	0.41
	1	181 (48)	167 (47)	
	2	167 (44)	163 (46)	
Respiratory infection <3 months^f^	+	229 (61)	206 (59)	0.43
Antibiotics <3 months^g^	+	53 (14)	58 (16)	0.39
PCV-13 vaccinated^h^	+	212 (56)	130 (37)	**<0.0001**
*S. pneumoniae* detected	+	212 (56)	178 (50)	0.13
VT pneumococci detected	+	18 (5)	44 (12)	**<0.001**
NVT pneumococci detected	+	185 (49)	137 (38)	**0.003**
*S. aureus* detected	+	29 (8)	41 (12)	**0.04**
NTHi detected	+	168 (45)	152 (43)	0.54
*M. catarrhalis* detected	+	183 (49)	188 (53)	0.38
Any of the bacteria detected^i^	+	317 (84)	290 (82)	0.55

**Abbreviations**: DC: day-care centre; PCV-13: the 13-valent pneumococcal conjugate vaccine; *S. pneumoniae*: *Streptococcus pneumoniae*; VT pneumococci: pneumococcal serotypes included in the PCV-13; NVT pneumococci: pneumococcal serotypes not included in the PCV-13; *S. aureus*: *Staphylococcus aureus*; NTHi: non-typeable *Haemophilus influenzae*; *M. catarrhalis*: *Moraxella catarrhalis*.

a. p-Value based on chi-square test for difference. Significant findings (p<0.05) in bold

b. Region: East (Tasiilaq, Kuummiut, Sermiligaaq, Kulusuk), West (Sisimiut, Sarfannguaq).

c. Having siblings attending a day-care institution.

d. Tobacco smoking inside the house.

e. Average number of persons per room: (0=less than 1 person per room in household), (1=1 to <2 persons per room), (2=≥2 persons per room), missing n=8 (2011) n=6 (2013).

f. Respiratory infections: any episode of rhinitis, acute otitis media, ear-discharge, tonsillitis or pneumonia within the last 3 months prior to nasopharyngeal sampling.

g. Having received treatment with antimicrobial drugs within the last 3 months.

h. PCV-13 vaccinated: vaccinated with ≥1 dose of the 13-valent pneumococcal conjugate vaccine.

i. Any bacteria: the detection of *S. pneumoniae*, *M. catarrhalis*, NTHi or *S. aureus* in the nasopharyngeal sample.

### S. pneumoniae

**Figure 1.**
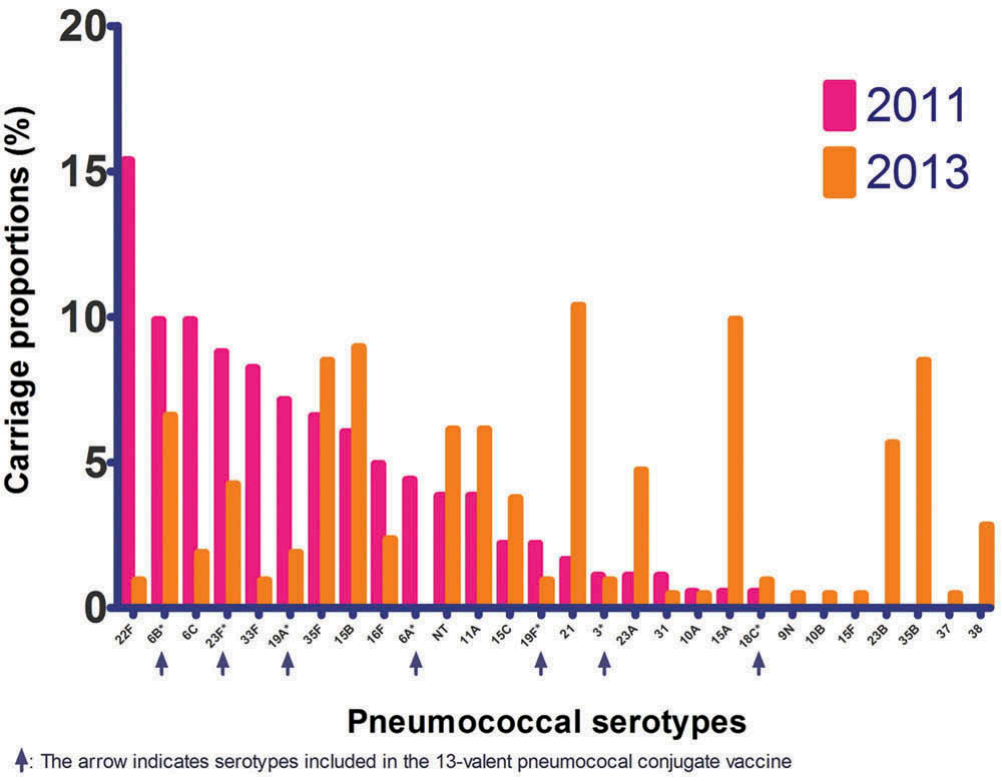
Nasopharyngeal pneumococcal serotype distribution among Greenlandic children aged 0–6 years in 2013 compared with serotype distribution in 2011 [12]. Arrows indicates serotypes included in the 13-valent pneumococcal conjugate vaccine.

**Figure 2.**
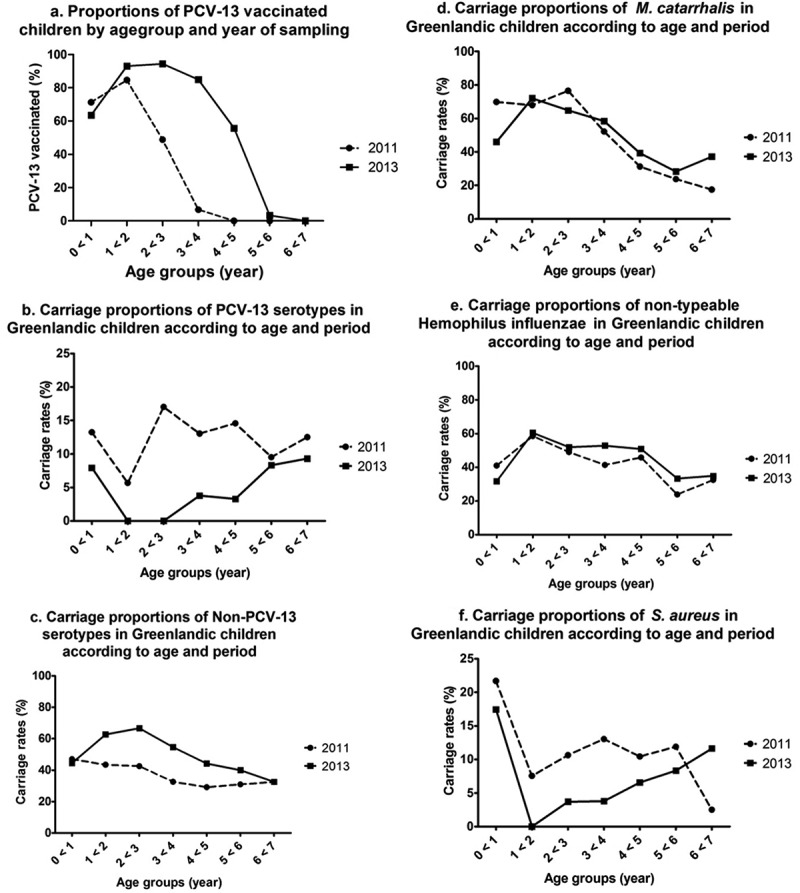
Proportions of PCV-13 vaccinated children by age group and year of sampling (a). Carriage pattern of *S. pneumoniae* (PCV-13 serotypes, non-PCV-13 serotypes) (b,c), *M. catarrhalis* (d), non-typeable *Hemophilus influenzae* (e), and *S. aureus* (f), according to age groups and period. Dotted lines represent results from a previous study conducted in 2011 [12].

The overall carriage rate of pneumococci in 2013 was 56%, and the most frequently identified serotypes were 6B, 11A, 15A, 15B, 21, 23A, 23B, 23F, 35B and 35F (Figure 1). In 2013, the overall pneumococcal carriage rate was basically unchanged compared with 2011 (Table 1). However, among PCV-13 vaccinated children carriage rates of VT pneumococci were significantly reduced (adjusted odds ratio (aOR) 0.43, 95% confidence interval (CI): 0.20–0.90) and carriage rates of NVT were significantly increased (aOR 1.63, 95% CI: 1.07–2.48) compared with unvaccinated children. Furthermore, when adjusting for PCV-13 vaccination, odds of carrying VT in 2013 were significantly reduced compared with 2011 (aOR 0.44, 95% CI: 0.24–0.82) whereas odds of NVT carriage were increased (aOR 1.36, 95% CI: 0.97–1.90) (Table 2). The reduction in VT carriage and increase in NVT carriage were predominantly seen among children aged 1–4 years, the group with greatest difference in vaccination rate (Figure 2(a)).

**Table 2.** Crude and adjusted odds ratios for bacterial nasopharyngeal carriage by *Streptococcus pneumonia*e, non-typeable *Haemophilus influe*nzae, *Moraxella catarrhali*s or *Staphylococcus aureu*s among Greenlandic children aged 0–6 years in 2013 compared with data from a cross-sectional study in 2011 [12].

	PCV-13				Year			
	No	Yes			2011	2013		
Bacterium	1 (ref.)	OR^a^ (95% CI)	aOR^b^ (95% CI)	p	1 (ref.)	OR^a^ (95% CI)	aOR^b^ (95% CI)	p
*S. pneumoniae*	1 (ref.)	1.31 (0.88–1.95)	1.19 (0.78–1.82)	0.41	1 (ref.)	1.23 (0.90–1.68)	1.18 (0.84–1.65)	0.33
VT	1 (ref.)	**0.44 (0.22–0.92)**	**0.43 (0.20–0.90)**	0.02	1 (ref.)	**0.42 (0.23–0.75)**	**0.44 (0.24–0.82)**	0.01
NVT	1 (ref.)	**1.65 (1.11–2.46)**	**1.63 (1.07–2.48)**	0.02	1 (ref.)	**1.45 (1.06–1.99)**	1.36 (0.97–1.90)	0.07
NTHi	1 (ref.)	1.17 (0.78–1.75)	1.29 (0.84–1.98)	0.24	1 (ref.)	1.07 (0.78–1.48)	1.10 (0.79–1.54)	0.58
*M. catarrhalis*	1 (ref.)	**1.72 (1.14–2.58)**	1.52 (0.99–2.33)	0.06	1 (ref.)	0.83 (0.60–1.16)	0.82 (0.58–1.16)	0.27
*S. aureus*	1 (ref.)	**0.51 (0.27–0.94)**	**0.48 (0.25–0.91)**	0.03	1 (ref.)	0.70 (0.42–1.18)	0.62 (0.35–1.07)	0.09
Any bacterium	1 (ref.)	1.58 (0.88–2.84)	1.43 (0.76–2.68)	0.27	1 (ref.)	1.13 (0.74–1.74)	1.04 (0.65–1.66)	0.87

**Abbreviations**: PCV-13: the 13-valent pneumococcal conjugate vaccine; OR: odds ratio; aOR: adjusted odds ratio; p: p-value for aOR; CI: confidence interval; *S. pneumoniae*: *Streptococcus pneumoniae*; VT: pneumococcal serotypes included in the 13-valent pneumococcal conjugate vaccine; NVT: pneumococcal serotypes not included in the 13-valent pneumococcal conjugate vaccine; NTHi: non-typeable *Haemophilus influenzae*;

*M. catarrhalis*: *Moraxella catarrhalis*; *S. aureus*: *Staphylococcus aureus*; Any bacterium: odds of carrying *S. pneumoniae*, NTHi, *M. catarrhalis* or *S. aureus* (grouped).

a. Odds ratios mutually adjusted for year of sampling and PCV-13 vaccination, as well as age groups (1 year intervals) and sex.

b. Additional adjustments: region (East/West Greenland), recent respiratory infection (otitis media, ear-discharge, nasopharyngitis, tonsillitis or pneumonia within the last 3 months), current day-care attendance (yes/no), having siblings in a day-care (yes/no) and ethnicity (“Inuit”, “mixed Inuit/other ethnicity” or “non-Inuit”).

c. Significant findings (p<0.05) in bold.

### NTHi

The average carriage rate among children <3 years was 47% (95% CI: 42–53%), and 40% (95% CI: 35–45%) among children ≥3 years. Overall, NTHi carriage remained stable during the period (Table 1, Figure 2).

### M. catarrhalis

Average carriage rate among children <3 years was 66% (95% CI: 61–71%) and 37% (95% CI: 32–41%) among children ≥3 years. Overall carriage rates were unchanged during the period (53% vs. 49%) (Table 1, Figure 2). In the crude analysis PCV-13 vaccination was associated with increased odds of *M. catarrhalis* carriage OR 1.72 (95% CI: 1.14–2.58), and when adjusted for confounders the association was attenuated although still indicating a positive association (aOR 1.52, 95% CI: 0.99–2.33).

### S. aureus

Among children less than 3 years average carriage rate was 12% (95% CI: 8–15%) and among children ≥3 years 8% (95% CI: 6–11%). Among vaccinated children there was a significant reduction in carriage rate of *S. aureus* (Table 2, Figure 2).

## Discussion

In line with previous PCV-7 carriage studies we found a substantial pneumococcal serotype shift from VT to NVT among vaccinated individuals 3 years after PCV-13 introduction in Greenland. In a double-blind randomised placebo-controlled trial, Obaro et al. [38] were among the first to report significant reductions in carriage rates of vaccine serotypes in Gambian infants vaccinated with a pentavalent PCV, counterbalanced by increasing carriage rates of NVT. In the following years several studies have confirmed these findings [24]. In our study we also observed a cohort effect showing reductions in VT carriage independent of PCV-13 vaccination, which may indicate herd immunity. Parallel to this, NVT carriage rates increased from 2011 to 2013 independently of PCV-13 vaccination. Besides the pneumococcal serotype shift, significant changes in carriage of other co-colonising clinically important pathogens were observed. *M. catarrhalis* carriage increased among PCV-13 vaccinated children. Natural fluctuations in rates of *M. catarrhalis* circulating in the community may explain this, but bacterial interactions may also be part of the explanation. We have previously reported a positive interaction between *M. catarrhalis* and NVT pneumococci [12]. Increase in NVT carriage following PCV-13 introduction may change the balance between the colonising bacteria in the nasopharynx towards higher carriage rates of *M. catarrhalis*. Whether increasing carriage rates of *M. catarrhalis* lead to changes in disease rates is not clarified. However, studies of PCV-7 impact on AOM have shown increases in proportions of *M. catarrhalis* and NTHi in middle ear fluid from PCV-7 vaccinated children with AOM, compared with non-vaccinated children, indicating an association [39–42]. A scenario of increasing carriage rates of *M. catarrhalis*, being an important oto-pathogen in young children [10], is concerning, especially since the Greenlandic population suffers from a very high burden of otitis media [4].

We also observed a significant reduction in carriage by *S. aureus* among PCV-13 vaccinated children. In a randomised controlled trial of the effect of PCV-7 on nasopharyngeal carriage, Van Gils et al. [43] found a negative association between pneumococci (both PCV-7 serotypes and non-PCV-7 serotypes) and *S. aureus*. The mechanism behind this association is unknown, but may include the production of hydrogen peroxide by pneumococci, which in vitro has been shown to eliminate *S. aureus* [44]. We found slightly higher rates of pneumococcal carriage among PCV-13 vaccinated children but the increase was not significant. The reduction in *S. aureus* carriage may also be related to the increase in *M. catarrhalis* carriage, since *S. aureus* and *M. catarrhalis* have previously been shown to be negatively associated [12]. One difference in the detection of *S. aureus* and the other bacteria is that the present study was not designed specifically to sample from the anterior nasal cavity where *S. aureus* resides. However, when entering the nasopharynx, the niche is passed twice, giving the opportunity to detect current *S. aureus* colonisation.

We believe a main strength of this study is the fact that it is based on two consecutive cross-sectional studies using exactly the same design, sampling method, transport medium and laboratory techniques, minimising study design bias. Furthermore, the study is population-based, based on random sampling from the CRS, a register updated on a daily basis minimising selection bias. We made a considerable effort in obtaining valid data from registers and questionnaires on potential confounders for carriage. However, some differences in baseline characteristics between the two cross-sectional studies were present, including proportions of children sampled in the two regions. Since the regions are separated by large distances local clustering in carriage proportions may have biased the results. However, we accounted for this by adjusting for region in the multivariable analyses. In 2013, the proportions of children attending a day-care centre and children being exposed to in-house smoking were smaller than in 2011. This could have created bias towards overestimating the effect of the vaccine, as both factors have been shown to increase the risk of *S. pneumoniae* carriage [45]. We did, however, include these factors in the multivariable analyses to limit the potential confounding effect.

A major limitation to our study was the lack of consistent pre-vaccination baseline data. Approximately one-third of the children had received the first dose of PCV-13 when the first cross-sectional study was conducted, which may have resulted in an underestimation of the direct vaccine effects. When including vaccination status in the multivariate regression analysis, we consistently found significant changes in carriage rates of VT and NVT independent of vaccination status according to the year of sampling (2013 compared with 2011), indicating a herd effect. Since our baseline data included a proportion of vaccinated children, we cannot rule out that the observed changes may to some extent have been biased by early indirect herd immunity effects.

Moreover, since we have only 3 years of study time post-PCV-13 introduction, we are unable to determine whether the observed changes reflect a transitional or steady state.

In conclusion, PCV-13 introduction in Greenland was associated with significant shifts in nasopharyngeal pneumococcal serotype distribution with marked reductions in VT carriage and increased carriage of NVT, while the overall carriage rate of pneumococci was unchanged. Independent of this, a period-specific reduction in carriage rates of vaccine-type pneumococci from 2011 to 2013 indicated a level of PCV-13-induced herd immunity. Furthermore, we found an increase in *M. catarrhalis* and reduction in *S. aureus* carriage among vaccinated children compared with unvaccinated children. Continued surveillance is warranted to clarify whether these changes are temporary or persist in the long term. It is also essential to monitor the clinical significance of these changes, including the invasive potential of the emerging NVT.
